# Novel Features and Abnormal Pattern of Cerebral Glucose Metabolism in Spinocerebellar Ataxia 19

**DOI:** 10.1007/s12311-018-0927-4

**Published:** 2018-03-12

**Authors:** Martin Paucar, Åsa Bergendal, Peter Gustavsson, Magnus Nordenskjöld, José Laffita-Mesa, Irina Savitcheva, Per Svenningsson

**Affiliations:** 1 Department of Clinical Neuroscience, Karolinska Institutet, Stockholm, Sweden; 2 Department of Neurology, Karolinska University Hospital, Stockholm, Sweden; 3 Department of Neuroimaging, Karolinska Institutet, Stockholm, Sweden; 4 Department of Clinical Genetics, Karolinska University Hospital, Stockholm, Sweden; 5 Department of Molecular Medicine and Surgery, Karolinska Institutet, Stockholm, Sweden; 6 Department of Nuclear Medicine, Karolinska University Hospital, Stockholm, Sweden

**Keywords:** Spinocerebellar ataxia types 19 and 22, *KCND3*, Channelopathy, [^18^F] FDG PET, White-matter abnormalities

## Abstract

Spinocerebellar ataxia type 19 (SCA19), allelic with spinocerebellar ataxia type 22 (SCA22), is a rare syndrome caused by mutations in the *KCND3* gene which encodes the potassium channel Kv4.3. Only 18 SCA19/22 families and sporadic cases of different ethnic backgrounds have been previously reported. As in other SCAs, the SCA19/22 phenotype is variable and usually consists of adult-onset slowly progressive ataxia and cognitive impairment; myoclonus and seizures; mild Parkinsonism occurs in some cases. Here we describe a Swedish SCA19/22 family spanning five generations and harboring the T377M mutation in *KCND3*. For the first time for this disease, ^18^F-fluorodeoxyglucose PET was assessed revealing widespread brain hypometabolism. In addition, we identified white matter abnormalities and found unusual features for SCA19/22 including early age of onset and fast rate of progression in the late course of disease in the oldest patient of this family.

**Electronic supplementary material:**

The online version of this article (10.1007/s12311-018-0927-4) contains supplementary material, which is available to authorized users.

## Introduction

Spinocerebellar ataxias (SCA) are a heterogeneous and growing group of autosomal dominant diseases enumerated in chronological order from SCA1 to SCA43 according to the current classification in use [1]. Pathological CAG expansions in different genes cause SCA1, SCA2, SCA3, SCA6, and SCA7 which represent up to 60% of all SCA cases; the remaining SCA subtypes are rare and in most cases associated with conventional mutations [2, 3]. Spinocerebellar ataxia types 19 (SCA19) and 22 (SCA22) are rare allelic channelopathies; linkage to chromosome region 1p21-q21 was reported first in two large Dutch and Chinese families [4, 5]; and the mutations in *KCND3*, which encodes the Shal-related voltage-gated potassium channel Kv4.3, associated with ataxia were discovered in 2012 by two independent research groups [6–8]. Previously, variants and mutations in *KCND3* were found to be associated with sudden unexpected death syndrome (SUDS) and later with Brugada syndrome-9 (BRGDA9) [9–11]. So far, only one ataxia patient harboring the mutation L450P in *KCND3* has been diagnosed with Brugada syndrome A. [10, 11]. Only 18 SCA19/22 families and sporadic cases of diverse ethnicities have been described so far [6, 7, 10–15]. In addition, two variants of unclear significance (VUS) associated with ataxia have been identified in a recent screening study [12]. The SCA19/SCA22 phenotype consists of adult-onset and slowly progressive cerebellar ataxia in most cases, frequent cognitive impairment and variable degree of myoclonus, polyneuropathy, and seizures. Mild Parkinsonism has been reported recently in two unrelated French SCA19 families [14]. The Thr377Met (T377M) mutation in *KCND3* has been described only once in a Japanese patient affected by pure cerebellar ataxia [7]. The aim of this study was to perform a comprehensive characterization of four affected members of a Swedish family spanning five generations; all affected family members harbor the T377M mutation in *KCND3*. This characterization includes clinical and cognitive evaluations, structural brain imaging and, for the first time for this disease, brain ^18^F-fluorodeoxyglucose PET ([^18^F] FDG PET).

## Materials and Methods

### Patients

**Fig. 1:**
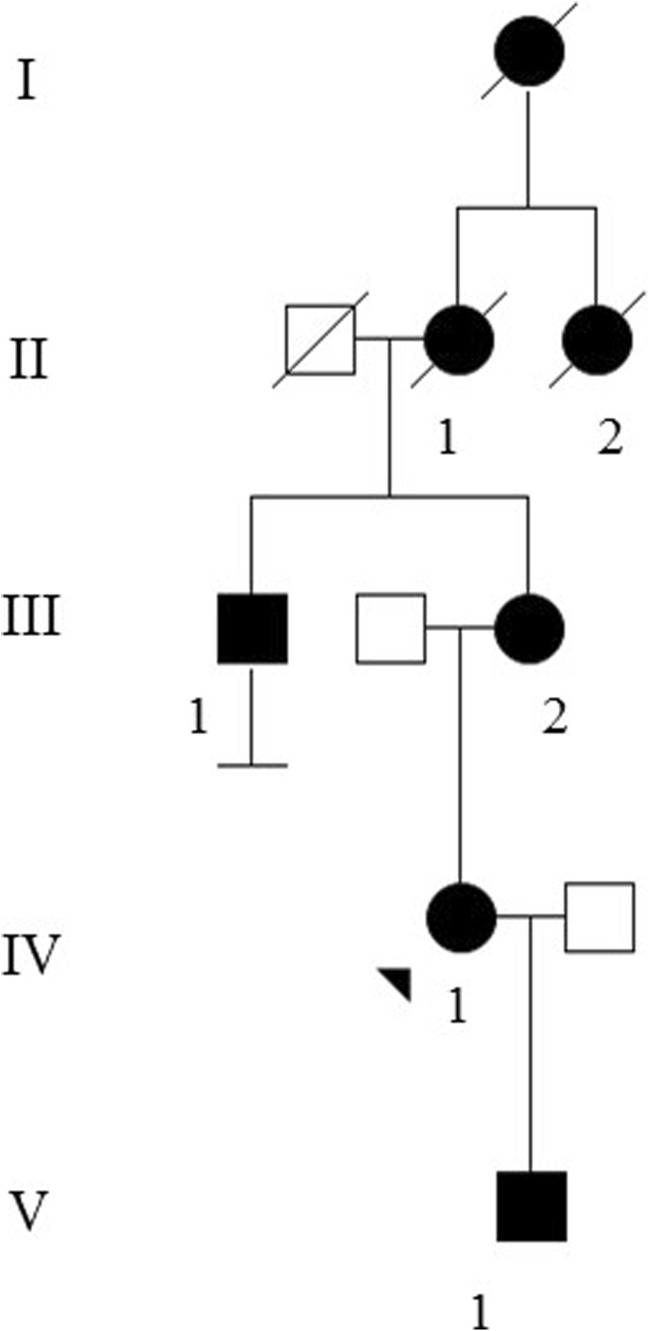
Pedigree of a Swedish SCA19/22 family. Disease status in generations I and II was asigned by history. All four tested patients (III:1, III:2, IV:1 and V:1) harbor the T377M mutation in *KCND3*.Variable degrees of axial ataxia, polyneuropathy, executive deficits, cerebellar atrophy and white matter abnormalities were identified

Four patients (III:1, III:2, IV:1, and V:1) from the same family of Swedish origin were recruited and underwent standard clinical investigation, psychometric testing, neuroimaging studies, electroneurography (ENeG) and genetic analyses. This study was carried out in accordance with the recommendations of the ethics committee in Stockholm and the radiation protection organization at the Karolinska University Hospital (Etikprövningsnämnden dnr 2016/2538-32) with written informed consent from all patients. All patients gave written informed consent in accordance with the Declaration of Helsinki. Disease status in three affected shown in the pedigree was assigned by history (Fig. 1). The following scales were used during the clinical evaluation: Assessment and Rating of Ataxia (SARA) and Inventory of non-ataxia Symptoms (INAS); neuropsychological testing of the index case III:1 (2015) and patient V:1 (2016) was carried out with the following batteries: (1) brief cognitive status: Montreal Cognitive Assessment (MoCA) and Mini Mental State Examination (MMSE); (2) general intellectual ability (IQ): Raven’s progressive matrices; (3) evaluation of verbal episodic memory: Rey Auditory Verbal Learning Test (RAVLT); (4) visuospatial episodic memory: Rey-Osterrieth Complex Figure Test (ROCFT); (5) working memory: digit span of the Wechsler Adult Intelligence Scale (WAIS-III); (6) spatial/visual construction: ROCFT, Copy and Block Design/WAIS; (7) verbal concept formation: similarities in WAIS-III; (8) word fluency: Controlled Oral Word Association Test (FAS/COWAT); (9) picture naming: Boston Naming Test (BNT); (10) information processing speed: Symbol Digit Modalities Test (SDMT); (11) Executive function: Trail Making Test, B (TMT); and (12) motor speed: finger-tapping test (FT), dominant and non-dominant hand. *Z* scores, computed on the basis of reference values from test manuals and handbooks, were used to compare results from various tests [16]; [17], a *z* score ≤ − 1.5 SD is compatible with a significant cognitive deficit.

### Imaging

Standard neuroimaging with 3T MRI scanners were performed in all four patients, an experienced neuroradiologist did a qualitative assessment. Brain metabolism was evaluated with [^18^ F] FDG PET in three patients at the Karolinska University Hospital in Huddinge. Imaging acquisition after intravenous injection of ^18^F-FDG (2 MBq/kg) was performed in a Biograph mCT PET/CT scanner (Siemens). A low-dose CT scan was used for attenuation correction of PET data. All appropriate corrections, including time of flow (TOF), were applied and reconstruction performed with OSEM (5 iterations, 21 subsets, 2.0 mm Gaussian filter). Visual analysis of PET scans was performed as well as semi-quantitatively using the syngo.via program. Comparison with healthy individuals was performed with three-dimensional stereotactic surface projection (3D-SSP) software using the whole brain as a reference region as well as an automated volume of interest (VOI)-based analysis of FDG uptake in the cortical and subcortical regions. None of the patients were treated with psychotropic medicines that may alter brain glucose metabolism.

### Genetic Analyses

Pathological nucleotide expansions for SCA1, SCA2, SCA3, SCA6, SCA7, SCA8 and dentatorubral-pallidoluysian atrophy (DRPLA) were ruled out first in the index case. In the same patient, a targeted high-throughput next-generation panel based on Illumina HiSeq platform was applied (CeGaT GmbH, Tübingen, Germany). This panel contained 300 genes of which the following are associated with ataxia: *ABCB7*, *ABHD12*, *ADCK3*, *AFG3L2*, *AMACR*, *ANO10*, *APTX*, *ATM*, *ATN1*, *ATP8A2*, *ATP1A3*, *ATXN1*, *ATXN2*, *ATXN3*, *ATXN7*, *ATXN10*, *BEAN1*, *C10orf2*, *CACNA1A*, *CCDC88C*, *COX20*, *CYP27A1*, *DARS2*, *DNMT1*, *EEF2*, *ELOVL4*, *ELOVL5*, *FGF14*, *FXN*, *GBA2*, *GJB1*, *GOSR2*, *GRID2*, *ITPR1*, *KCNC3*, *KCND3*, *KIF1C*, *MRE11A*, *MTPAP*, *MTTP*, *NOP56*, *NPC1*, *NPC2*, *OPA1*, *OPA3*, *PDYN*, *PEX7*, *PHYH*, *PNPLA6*, *POLG*, *PPP2R2B*, *PRKCG*, *SACS*, *SETX*, *SIL1*, *SPG7*, *SPTBN2*, *STUB1*, *SYNE1*, *TBP*, *TDP1*, *TGM6*, *TTBK2*, *TTPA*, *VAMP1*, *VLDLR*, *WWOX*, and *ZNF592*. Segregation was performed after a variant in *KCND3* was identified in the index case.

## Results

### Clinical Findings

The clinical features are summarized in Table 1. Age of onset was not possible to determine in the index case (IV:1) who is now 45 years old. She reported clumsiness since early childhood with clear difficulties to perform balance exercises in school. Since age 23 years, the patient has reported a clear progression but was not referred to us until age 40 years. During this period, her speech had become slurred and the patient experienced numbness in her extremities; neurophysiology was normal in two occasions, nevertheless. Patient III:2 is 65 years old; she never sought medical care for her ataxia. She presented with a recurrent no-no head tremor for many years and clumsiness since childhood. Similar to her daughter, she has been unable to perform tandem gait since early age. The exact age of onset was not possible to determine; however, the patient reports unsteadiness for as long as she can remember. Her past medical history (PMH) consisted of type 2 diabetes (T2DM) and hypertension. The rate of ataxia progression was very slow; however, during the last 12 years, she reported several falls and suffered from limb fractures in four different occasions. Last year, she was diagnosed with osteoporosis and started treatment with a bisphosphonate. Increased threshold for cold was found suggesting incipient small fiber neuropathy but the remaining neurophysiology was otherwise normal. Patient III:1 reported a similar age of onset with very slow progression rate. However, ataxia in this 78-year-old man has progressed quickly during the last 2 years. His comorbidities include chronic conditions like T2DM, obesity (BMI = 31), and asthma. Right kidney cancer was diagnosed at age 76 years and motivated nephrectomy; since then, the patient has been on dialysis. Last year, he was admitted to hospital for acute abdominal pain. Intestinal ischemia due to an episode of paroxysmal atrial flutter was diagnosed which necessitated surgery. A severe dysphagia became evident after this procedure, and the patient needed a percutaneous endoscopic gastrostomy (PEG) for 5 months. He is now wheel chair-dependent but is still able to navigate it. Neurological findings at examination included severe ataxia, areflexia, nystagmus, restricted vertical gaze, and increased muscle tone in the left arm. Neurophysiological tests revealed a length-dependent sensory axonal polyneuropathy. The youngest patient (V:1) is 21 years old; he was healthy and able to play American football until age 18 years. At this point, he started to experience truncal tremor and gait difficulties. His tremor was alleviated with ondansetron. His ataxia is mainly axial and has not progressed in the course of 2 years. Besides dysmetria, nystagmus and hypometric saccades were evident upon examination. Reflexes and neurophysiological tests were normal (Table 1).

**Table 1:** Clinical features found in a Swedish SCA19/22 family, axial ataxia predominates. None of the patients has Brugada syndrome. Functional stage (0–6) from the Friedreich’s ataxia rating scale (FARS)

Patient	Age of onset	SARA at first exam (age)	SARA at latest exam (age)	INAS at last exam	Reflexes	ENeG	Functional stage	Eye mov.	Comorbidity	Cognitive assessment	Structural imaging	Reduced metabolism on FDG-PET
III:1	18	18 (73)	24 (78)	5^a^	Aref.	PNP	5	Nyst SNP Rigd^b^	T2DM, HT, obesity, kidney cancer and failure, AF, MD, hearing impairment^c^, osteoarthritis, asthma	MoCA = 15 p	Moderate vermis atrophy and WMA	NA
III:2	Childhood	4 (63)	6 (65)	2	Aref.	SFN	2	Nyst.	T2DM, HT, myopia	MoCA = 24 p	Mild vermis atrophy and WMA	PFC Motor cortex
												Temporal cortex
												Vermis
IV:1	Childhood	8 (43)	8 (45)	2	Red.	N	2	Nyst	Diplopia due to esophoria	Executive deficits	Mild vermis atrophy and WMA	PFC and parietal regions
										MoCA = 25 p		Thalamus
												Entire cerebellum
V:1	18	*5 (19)*	5 (21)	2	N	N	1	Nyst	None	Executive deficits	Moderate vermis atrophy	Temporal and parietal regions
										MoCA = 27 p		

*A* absent, *AF* atrial flutter, *Aref* areflexia, *Eye* mov eye movements, *HT* hypertension, *INAS* inventory of non-ataxia signs, *MD* macular degeneration (right eye), *MoCA* Montreal cognitive assessment, *N* normal, *NA* not assessed, *Nyst* nystagmus, *PFC* prefrontal cortex, *PNP* polyneuropathy, *Red* reduced, *Rigd* rigidity, *SARA* scale for the assessment and rating of ataxia, *SFN* small fiber neuropathy, *SNP* supranuclear palsy, *T2DM* type 2 diabetes mellitus, *WMA* white matter abnormalities

^a^Patient III:1 has a left side rigidity and significant comorbidity, he is confined to a wheel chair, all the other are ambulatory without assistance

^b^Eye movement abnormalities in the index case and patient III:2: broken smooth up pursuit, nystagmus and hypometric saccades. III:2. Patient III:1 displays also partial restriction of vertical gaze, absence of vertical optokinetic nystagmus suggests SNP. Patient V:1: has nystagmus and hypometric saccades

^c^This reduction was mild and non-progressive, found at age 46 years

Patients III:1 and III:2 had limited school attendance. Patient III:1 went to elementary school for 7 years while patient III:2 went to a vocational school (2 years) after 8 years of elementary school. Patients III:1 and III:2 declined psychometric testing for the purpose of this study. Patient III:1 had a MoCA score of 15points with major deficits found in visuospatial/executive tasks as well as in attention and delayed recall. Patient III:2 obtained a MoCA score of 24; this examination revealed mainly deficits in attention. Patient IV:1 has a total education of 13 years and has been working as a nursing assistant, her score was 25. Patient V:1 has totally 12 years of education, and in the last 2 years in school, he went through a vocationally oriented program preparing for work with children and youth. A brief cognitive examination with MMT yielded an average result (28 points) while his MoCA score was 22 which is significantly beneath the normal range. The cognitive deficits in patient IV:1 were mild and non-significant. On the other hand, patient V:1 had a significantly low performance in tests assessing visuospatial episodic memory (ROCFT), working memory (Digit span/WAIS), picture naming ability (BNT), word fluency (FAS/COWAT), and executive function/simultaneous capacity (TMT B). In contrast, the result for verbal episodic long term memory was significantly above average (RAVLT/retention). The cognitive features are summarized in Table 2.

**Table 2:** Summary of cognitive features in two patients from a Swedish SCA19/22 family; patients III:1 and III:2 had MoCA scores of 15 respectively 24 but declined psychometric testing. A *z* score ≤ − 1.5 SD is compatible with a significant deficit (*)

Cognitive domain	Neuropsychological test	Patient IV:1	Patient V:1
		2015 (*z* score)	2016 (*z* score)
Brief cognitive status examination	MoCA (Montreal cognitive assessment)	25 (− 1.09)	22 (− 2.45)*
	MMT (mini mental test)	NA	28/30
General intellectual ability IQ	Raven’s progressive matrices	NA	125 (1.70)
	Matrices WAIS	12 (− 1.0)	NA
Verbal episodic memory	RAVLT (Rey Auditory Verbal Learning Test) learning	56 (0.76)	59 (0.93)
	RAVLT retention	12 (0.59)	15 (1.67)
Visuospatial episodic memory	ROCFT (Rey-Osterrieth Complex Figure Test) immediate recall	18 (− 0.7)	11.5 (− 3)*
	ROCFT (delayed recall)	19.5 (− 0.4)	10.5 (− 3)*
Working memory	Digit span/WAIS	13 (− 1)	10 (− 1.67)*
Spatial/visual construction	ROCFT copy	31 (− 0.59)	31 (− 0.59)
	Block design/WAIS	28 (− 1.0)	37 (− 0.33)
Verbal concept formation	Similarities/WAIS	22 (0)	19 (− 0.67)
Word fluency	FAS/COWAT (Controlled Oral Word Association Test)	31 (− 0.89)	22 (− 1.73)*
Picture naming ability	BNT (Boston Naming Test)	NA	38 (− 6.39)*
Information processing speed	SDMT (Symbol Digit Modalities Test)	NA	56 (− 0.74)
	Digit symbol/WAIS	44 (− 1.33)	NA
Executive function	TMT B (Trail Making Test)	NA	94 (2.22)
Motor speed	FT (finger-tapping test) dominant hand	NA	52 (0.38)
	FT non-dominant hand	NA	45 (− 0.36)

*NA* not assesed

None of the four patients had signs of Brugada syndrome or ventricular arrhythmia; there were no cases of SUDS in this family either. Recently, patient III:1 was found to have a bifascicular block, a long-term registration with an ambulatory electrocardiography device has been ordered and the patient has been referred for a new cardiac risk stratification. A previous long-term registration yielded normal results.

### Structural Brain Imaging and [^18^F] FDG PET

**Fig. 2:**
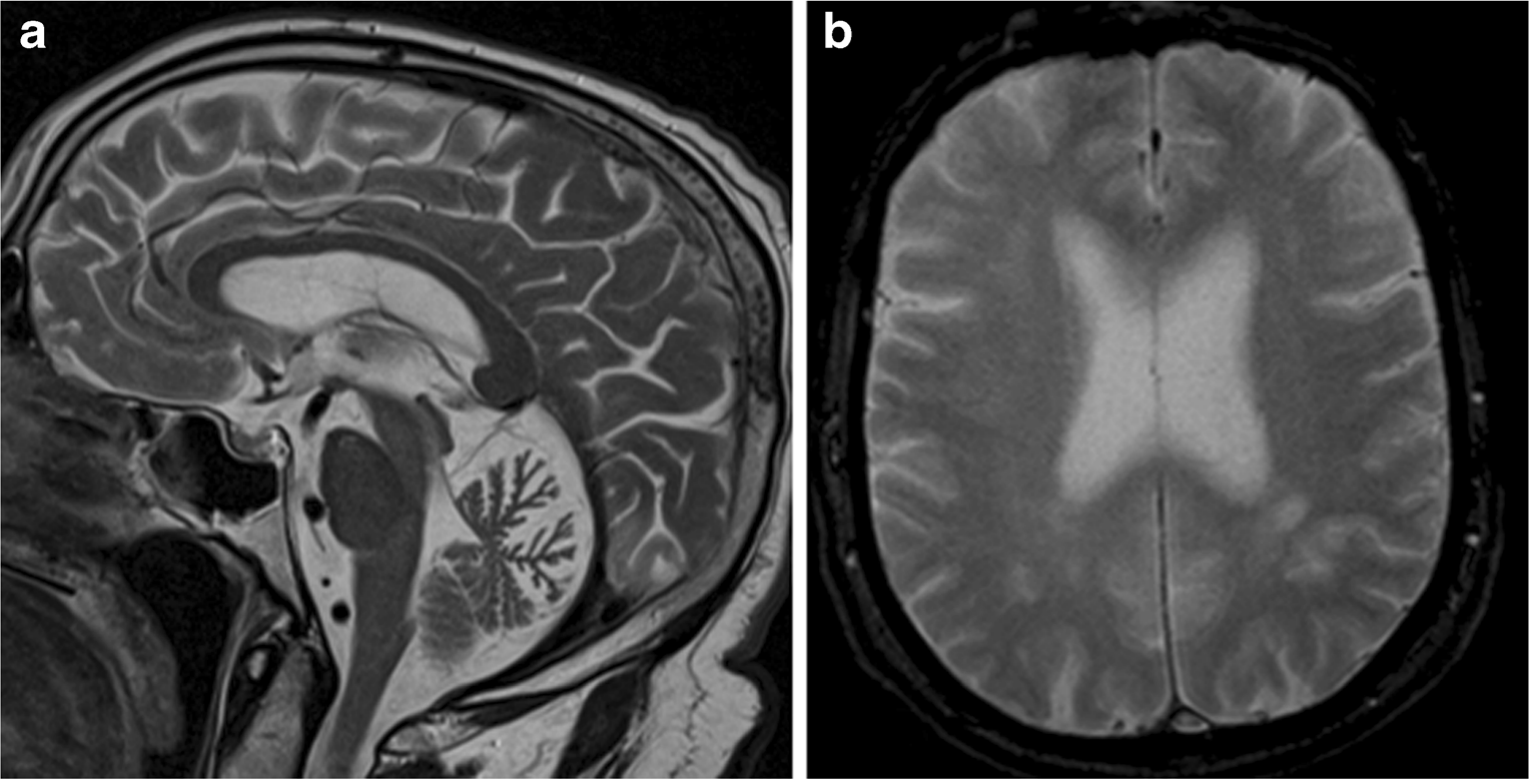
Brain MRI of patient III:1. **a** Midsagittal T2-weighted image displays moderate vermis atrophy. **b** Coronal T2-weighted image showing periventricular and deep white matter hyperintensities

**Fig. 3:**
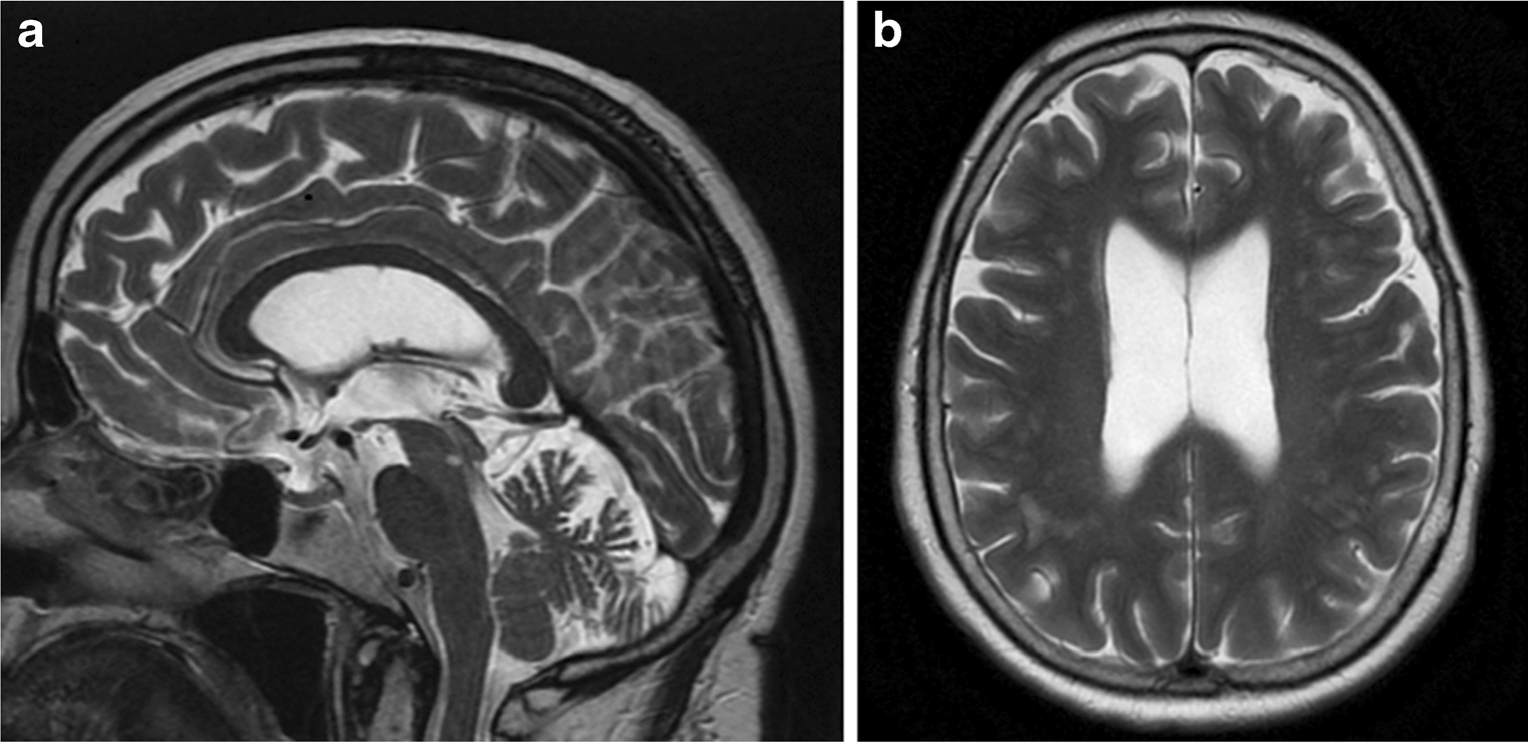
Brain MRI of patient III:2. **a** Mild vermis atrophy is evident on this midsagittal T2-weighted image. **b** Coronal T2-weighted image displays deep white matter hyperintensities

**Fig. 4:**
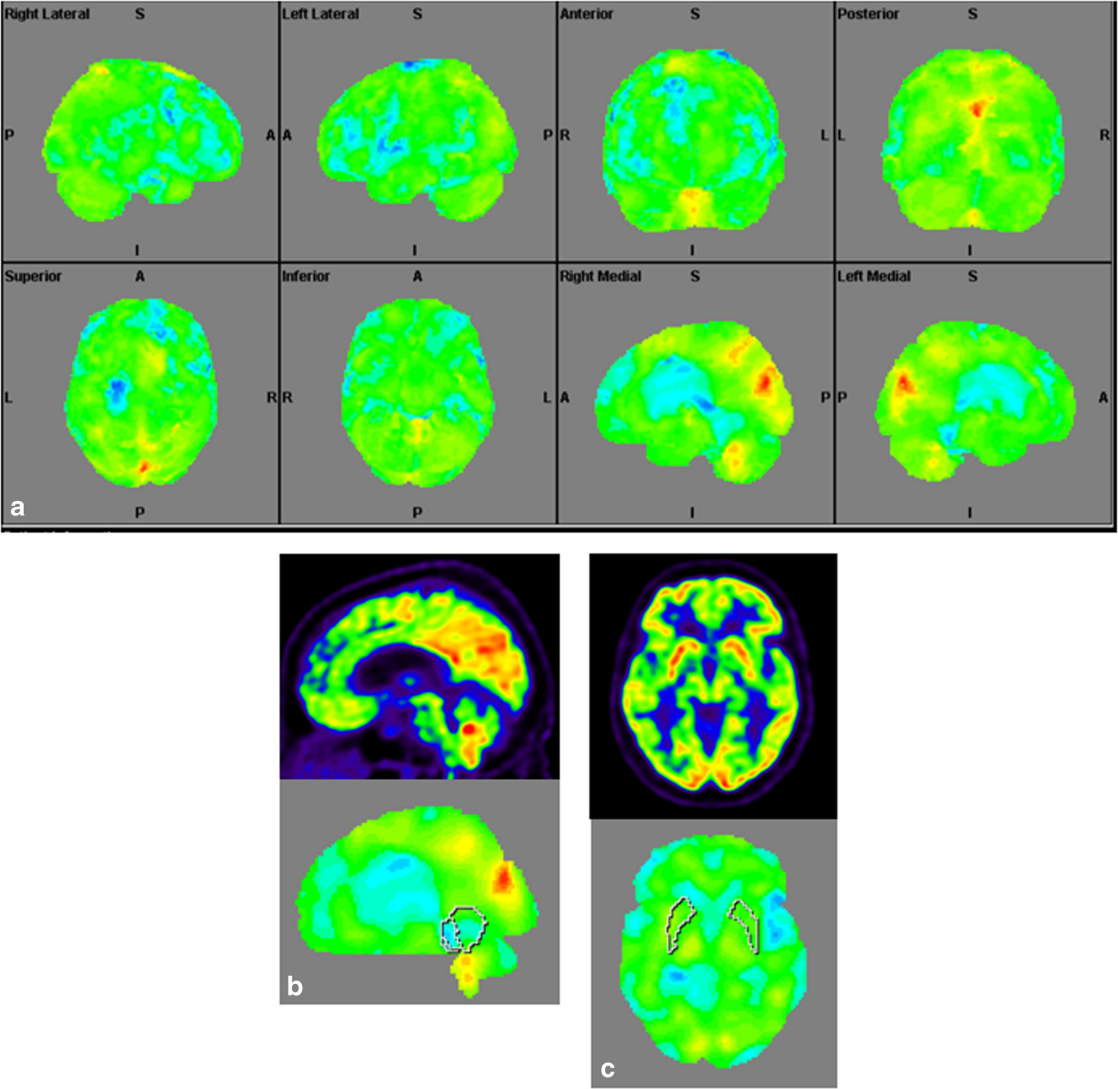
Surface projections (3DSSP) of brain [^18^F] FDG PET findings for patient III:2. **a** Widespread hypometabolism in the prefrontal cortex and some areas of the motor and temporal cortices is evident. Metabolism is also reduced in the vermis but normal in the cerebellar hemispheres. **b** Midsagittal image shows reduced FDG uptake in the vermis (upper case) as compared to the reference values in VOI templates (lower case). **c** Coronal image shows normal FDG uptake in the putamen (upper case) as compared to the reference values (lower case)

**Fig. 5:**
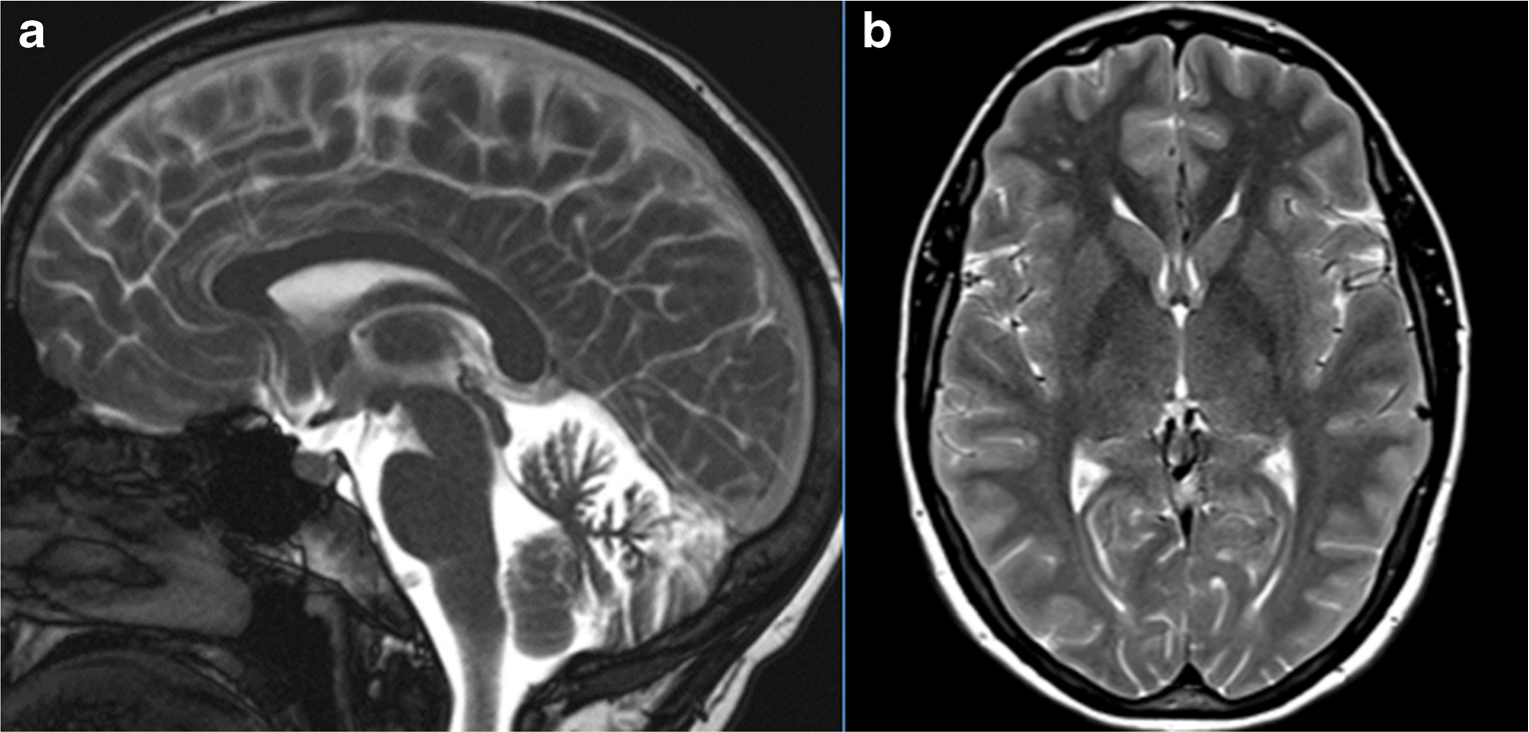
Brain MRI of patient III:2. **a** Midsagittal T2-weighted image shows mild vermis atrophy. **b** Punctate white matter hyperintensities in the frontal lobe are evident in the coronal T2-weighted image

**Fig. 6:**
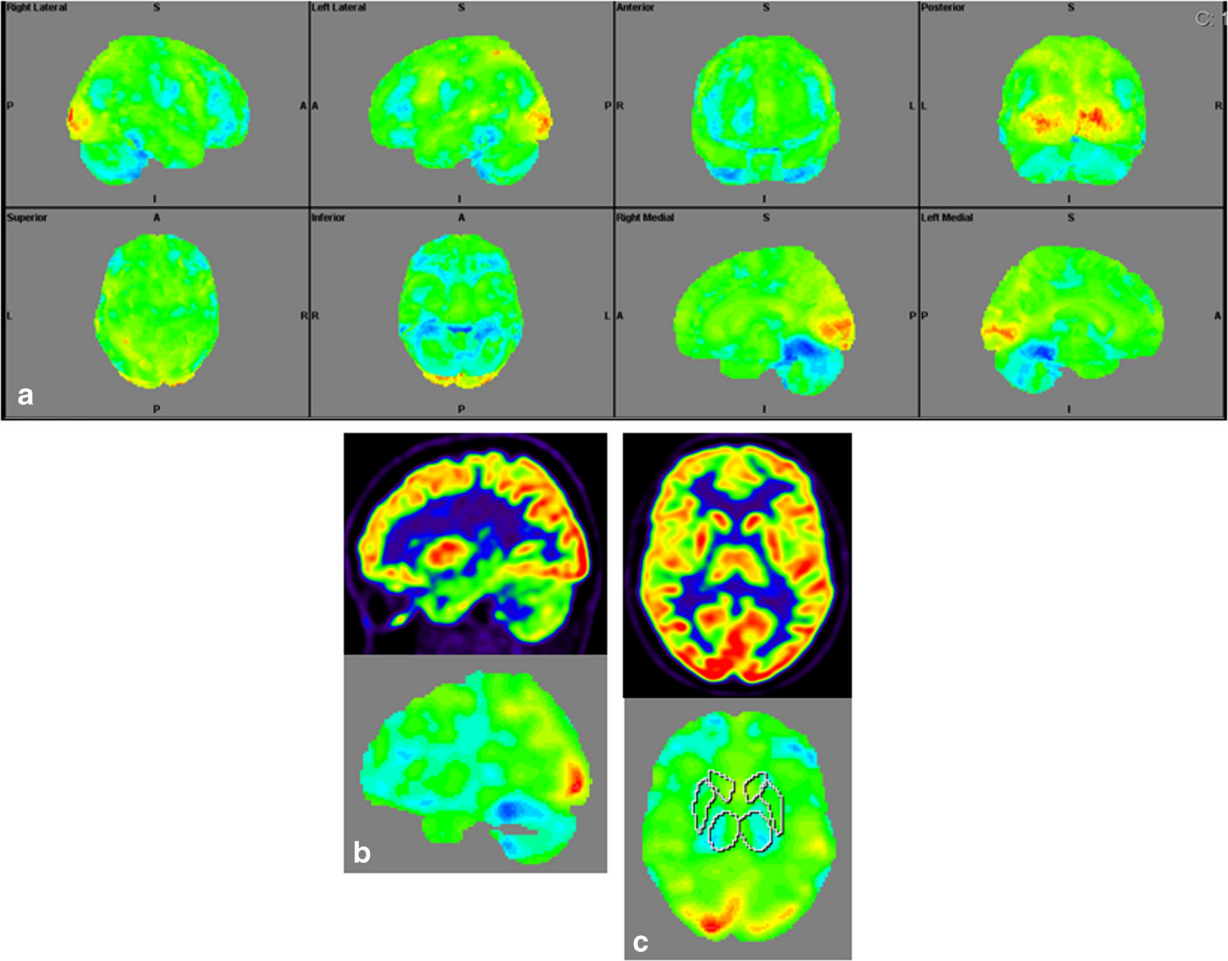
Surface projections (3DSSP) of brain [^18^F] FDG PET findings for patient IV:1. **a** Metabolism is reduced in the prefrontal and parietal cortex as well as in the entire cerebellum. **b** Midsagittal image shows reduced FDG uptake in the cerebellum (upper case) as compared to the reference values in the VOI templates (lower case). **c** A similar abnormality is evident in the thalami on the coronal image (upper case) as compared to the reference values (lower case), FDG uptake in the putamen is otherwise normal

**Fig. 7:**
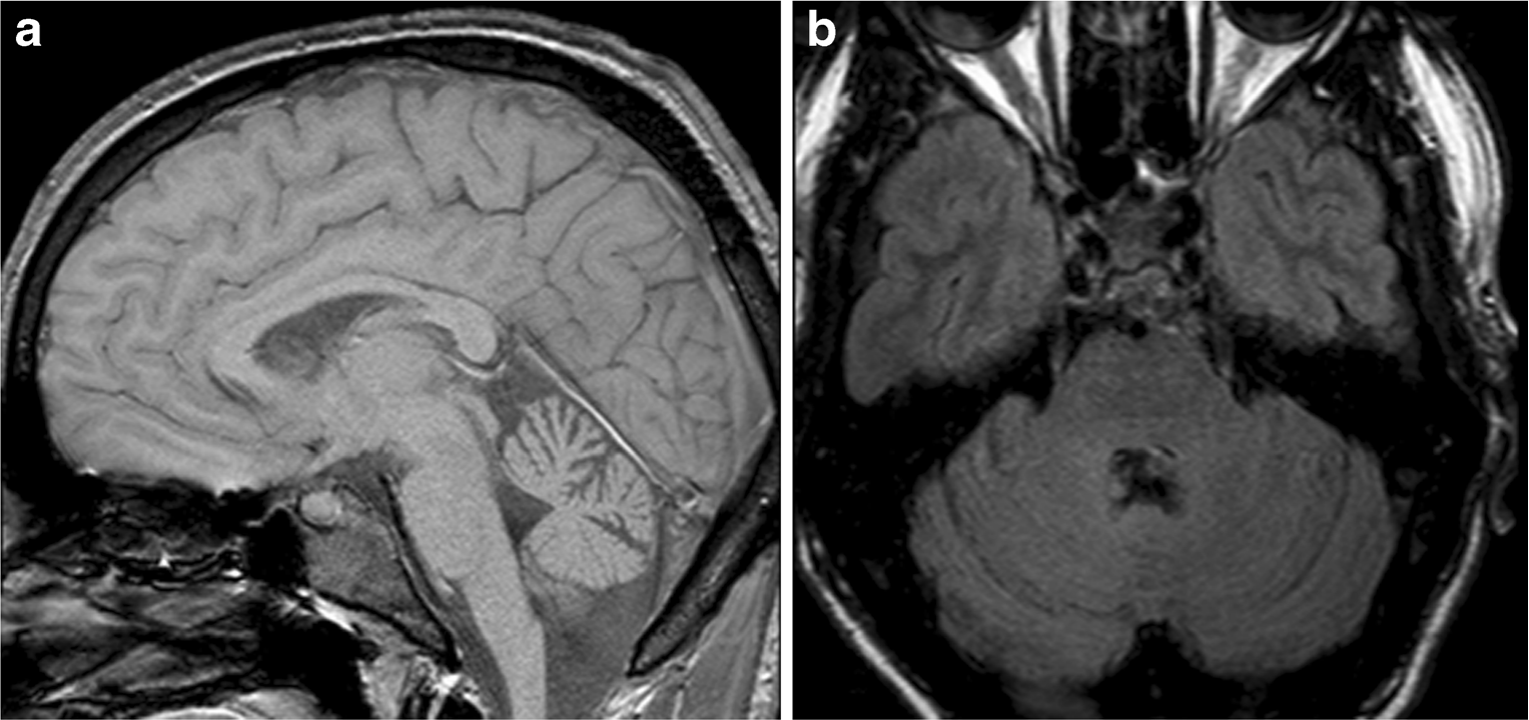
Brain MRI of patient V:1. **a** Midsagittal and coronal T1-weighted image. **b** Mild cerebellar atrophy

**Fig. 8:**
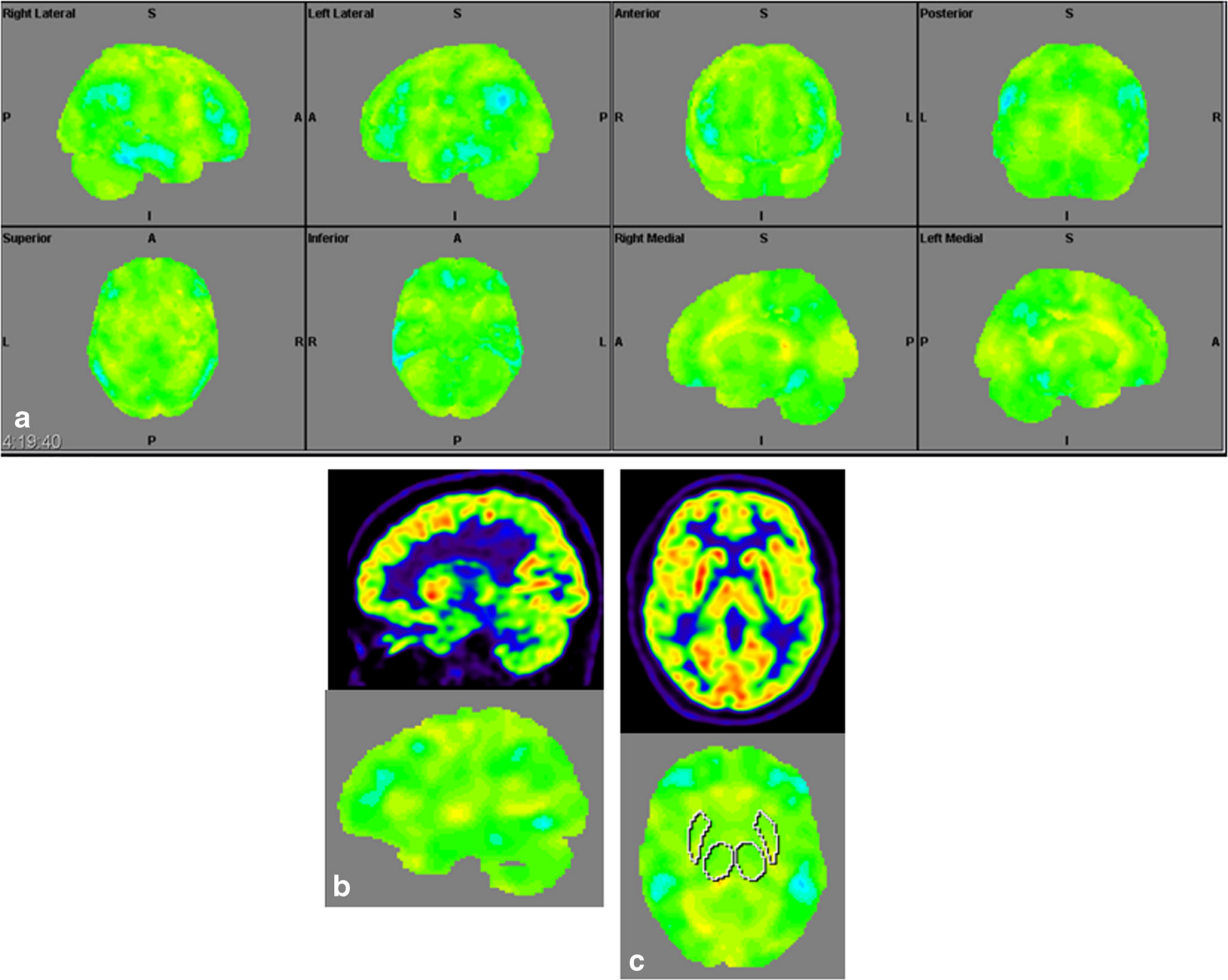
Surface projections (3DSSP) of brain [^18^F] FDG PET findings for patient V:1. **a** Displays hypometabolism in the prefrontal cortex, lateral temporal cortex, and in some areas of the parietal cortex. **b** Metabolism in the cerebellum is normal on this midsagittal image (upper case). **c** FDG uptake is also normal in the basal ganglia and thalami on the coronal image (upper case); reference values in VOI templates are displayed in the lower cases of B and C

The imaging data are summarized in Table 1 and are shown in Figs. 2, 3, 4, 5, 6, 7, and 8. Brain MRI revealed atrophy of the vermis and supratentorial white matter hyperintensities (WMH) in patients III:1 and III:2 and to a lesser degree in the index case (Figs. 2, 3, and 5). These WMH were particularly widespread in III:1 (Fig. 2). The index case was not affected by any other comorbidity usually associated with WMH, suggesting that these abnormalities may be an underlying feature of SCA19/22. WMH were not evident in the youngest patient (V:1). [^18^F] FDG PET revealed hypometabolism in the entire cerebellum, thalamus, prefrontal cortex (PFC), and parietal regions of the index case (IV:1) (Fig. 6). Her mother had reduced glucose metabolism not only in the PFC but also in the vermis as wells as in the motor and temporal cortex (Fig. 4). The youngest patient (V:1) had hypometabolism in the PFC, parietal, and inferior temporal regions but surprisingly not in the cerebellum (Fig. 8). In the latter structure, atrophy of the vermis was evident (Fig. 7).

### Genetic Analyses

The heterozygous T377M (c.1130C>T) mutation in *KCND3* was identified in the index case first and confirmed by Sanger sequencing. The other affected in the family (III:1, III:2, and IV:1) were found to harbor this mutation. T377M is located in a highly conserved amino acid in exon 3 that is predicted to be pathogenic by three different programs (Mutation Taster, PolyPhen-2 and SIFT). In addition, this variant is absent in ExAC and Swedgene databases. The latter contains genome data on 1000 subjects. Taken together, the T377M is now a class 5 variant according to current criteria proposed by the American College of Medical Genetics [18].

## Discussion

Our findings cement the T377 variant as a causative mutation for SCA19/SCA22. This is the first time that functional imaging data is reported in SCA19/22; also new is the ethnic background of this family. Ataxia channelopathies are in general characterized by early motor onset, intellectual disability and slow disease progression as demonstrated in a recent genetic screening performed in a large European ataxia cohort [12]. Most of our findings are in line with those conclusions. In the aforementioned screening which included 412 patients, only one clear pathogenic variant and two variants of unclear significance (VUS) in the *KCND3* gene were identified. [12]. In a large cohort of 1500 ataxia patients in the UK, only three SCA19/SCA22 patients were identified while a previous screening in a smaller Asian cohort yielded negative results [13, 19]. Penetrance of *KCND3* mutations associated with ataxia is very high as shown in the Swedish family; reduced penetrance has been described only once for *KCND3* mutations (M373I) [6].

The spectrum of *KCND3* mutations constitutes a unique cardiocerebral syndrome; *KCND3* is the only known ataxia gene associated with cardiac arrhythmia so far. Besides the described associations with SUDS and Brugada syndrome, a recent association with atrial fibrillation has been reported [20]. Accordingly, the *KCND3* gene is widely expressed; higher levels of expression are found in the cerebellum and in the heart [21–23]. Despite the importance of voltage-gated K^+^ (Kv) channels for action potential repolarization in cardiomyocytes, *KCND3 knockout* mice do not differ from wild type animals at least in terms of survival as well as for structural and electrophysiological properties of cardiomyocytes [24]. To our knowledge, there is no published data on any potential structural or functional cerebellar abnormalities in this *knockout* model. Of all the four mutations or variants in *KCND3* associated with Brugada syndrome or SUDS, three are located in the C-terminal and one in the S6 transmembrane domain A. [9–11]. This scarcity of cases precludes the delineation of clear genotype-phenotype correlations at the moment; however, risk stratification for cardiac arrhythmia is warranted in patients with *KCND3* mutations regardless of the mutation type or location within the gene. Other familial potassium channelopathies associated with cerebellar dysfunction include spinocerebellar ataxia type 13 (SCA13), episodic ataxia type 1 (EA1), and sensorineural deafness, ataxia, mental retardation, and electrolyte imbalance (SESAME syndrome) which are caused by mutations in the *KCNC3*, *KCNA1*, and *KCNJ10* genes, respectively [25–27]. Different to SCA13 in which some patients have a non-progressive course [28], SCA19/22 is clearly progressive [6, 7]. Interestingly, an association between epilepsy and long QT syndrome (LQTS) has been proposed for mutations in *KCNQ1*; however, ataxia is, to the best of our knowledge, not part of the disease spectrum associated with *KCNQ1* mutations [29]. Ataxia is on the other hand one of several acquired autoimmune disorders in the spectrum associated with antibodies to voltage-gated potassium channel (VGKC) which also include Morvan’s syndrome, neuromyotonia, limbic encephalitis, and cardiac arrhythmia [30–32].

All the four patients in the Swedish SCA19/22 family display atrophy of the vermis and three of them display varying degrees of white matter hyperintensities (WMH). WMH are common findings among the elderly and of vascular origin; these abnormalities are associated with cognitive decline particularly when they are progressive [33, 34]. Different to the previously described SCA19/22 families, we did not find clear atrophy in the cerebellar hemispheres or the less common cerebral atrophy [4, 35]. In contrast to a Dutch SCA19 family, we did not find evidence of myoclonus nor psychiatric manifestations (e.g., impulsivity or depression) [36]. Onset in the index case and patient III:2 in the Swedish family was during early childhood, but the exact timing was not possible to determine. Early onset is in fact very rare for SCA19/22, but slow disease progression is common [15, 36]. At least in one of the patients (III:1), there was evidence of global cognitive impairment as evaluated by MoCA. These deficits were predominantly in visuospatial and executive tasks (IV:1 and V:1) and are similar to what have been reported in two unrelated French families [14]. However, the French families display a more complex syndrome that includes Parkinsonism, seizures, as well as psychiatric symptoms. Previously, impairment in the Wisconsin Cart Sorting Test (WCST) suggesting impaired ability for abstract thinking and set-shifting was identified in SCA19 [14, 37, 36]. Similar deficits have been found in some of the polyglutamine SCAs (SCA1, SCA2, SCA3, and SCA6) [38, 39]. The cognitive deficits we have found in this family and their pattern of brain glucose metabolism support the notion that a frontal-executive dysfunction occurs in SCA19 [36]. It is noticeable that the general intellectual ability was not low neither in patient IV:1 nor V:1. However, the contrast between patient IV:1 whose cognitive deficits are rather mild and the pronounced dysfunction in several cognitive domains found in patient V:1 is striking. Mild deficits are hard to reconcile with the widespread brain hypomethabolism in patient IV:1. The reasons for this remarkable intrafamilial variability are unknown at the moment. The main limitations of this study are small sample size and the fact that neuropsychological testing and [^18^F] FDG PET were performed at different time points.

Reasonably, significant comorbidity contributed to the faster rate of motor progression and low MoCA score in the oldest patient (III:1) in the Swedish SCA19/22 family. Both T2DM and kidney failure are well-known risk factors for cognitive decline and dementia [40, 41]. Differently to the described Japanese SCA19/22 patient harboring the T377M mutation in *KCND3*, we found polyneuropathy in two patients from the Swedish family who were also affected by T2DM. However, this feature has to be interpreted with great caution since damage to periphery nerves is commonly associated with T2DM. Nonetheless, it will be of value to learn more about non-ataxia features in relatives to the Japanese patient harboring the T377M mutation [7].

Few PET studies have been performed in SCA; so far, only two small [^18^F] FDG PET studies on ataxia channelopathies have been published [42, 43]. The pattern of hypometabolism we described in SCA19/22 is reminiscent of the abnormal pattern found mainly in SCA6 and to some degree in SCA2 [43]. In SCA6, a calcium channelopathy, reduced glucose metabolism was found in the cerebellum and frontal and prefrontal cortices [43]. Different to SCA3 we did not find any evidence of abnormal metabolism in the basal ganglia. Our findings regarding variable brain hypometabolism are not specific enough to draw definitive conclusions. Additional studies are needed in order to assess the pattern of FDG metabolism in patients with other *KCND3* mutations.

Only one SCA19/22 neuropathological assessment has been reported so far; besides moderate to severe loss of Purkinje cells, reduced levels of KCND3 protein were found in cerebellar homogenates [44]. Also similar to SCA6, some neuronal loss was identified in a few brainstem nuclei [3, 45]. Some of the mutant Kv4.3 proteins remain trapped in the endoplasmic reticulum leading to a loss of function [6, 7]. Later, evidence of a dominant negative effect in SCA19/22 was provided for some mutations [10, 11] which is similar to previous findings in EA1 and SCA13 [26, 27, 46, 47]. Despite these important advances, many aspect of the disease remain to be explored. For instance, long-term follow-up studies are needed to determine the prevalence and risk of cardiac arrhythmia. Additional neuropathological studies of SCA19/22 are needed in order to establish genotype-phenotype and clinic-pathological correlations. Finally, the potential pathogenic role of the two recently identified VUS in *KCND3* [12] and the impact of potential gene modulators on disease expressivity remain also to be investigated.

## Electronic Supplementary Material

Supplementary material Variable degree of ataxia, nystagmus and dysarthria are evident in three patients with the T377M mutation in *KCND3,* these recordings made in 2014 and 2015. The oldest patient in this kindred had the most severe motor features; axial ataxia predominates but variable dysmetria is also evident in all the three patients (the youngest patient is not shown). (MP4 272494 kb)
